# Effects of L-theanine on cognition, mood and sleep: evidence from human trials

**DOI:** 10.3389/fnut.2026.1922819

**Published:** 2026-09-04

**Authors:** Jack Cotter, Hannah E. Kilpatrick

**Affiliations:** Research and Development, The Water Street Collective Ltd, Southampton, United Kingdom

**Keywords:** caffeine, cognition, L-theanine, mood, review, sleep, stress, supplement

## Abstract

L-theanine is a naturally occurring, non-proteinogenic amino acid found in the tea plant *Camellia sinensis* and some mushrooms. The widespread consumption of tea, together with its long history of traditional use for supporting various aspects of wellbeing, has led to significant scientific interest in the effects of L-theanine. Much of this research has focused on L-theanine's ability to modulate the central nervous system and related functions. This has also led to its inclusion in dietary supplements for its purported effects on human health and performance. In this mini-review, we summarize the latest evidence on L-theanine and its effects on cognition, mood and sleep based on systematic reviews and meta-analyses of human randomized controlled trials. We also examine evidence relating to its safety, consider limitations of the existing scientific literature and highlight key areas for future research. Current evidence in healthy adults suggests that supplementation with 100–400mg of L-theanine can produce small but statistically significant improvements in aspects of cognitive performance, particularly on visual attention tasks and when combined with caffeine. It has also been reported to reduce both perceived and physiological markers of stress and anxiety under stress-inducing conditions at doses of 200–400mg/day, without causing sedation. Additionally, trials report that supplementation with 200–450mg/day of L-theanine can support sleep quality, with little effect on total sleep duration. Supplementation with L-theanine has generally been reported to be well-tolerated in clinical trials. Further high-quality studies are warranted to establish optimal dosing strategies, clarify underlying mechanisms and evaluate potential interactions with other bioactive compounds.

## 1 Introduction

L-theanine is a naturally occurring, water-soluble, non-proteinogenic amino acid that can be found in the tea plant *Camellia sinensis* and some mushrooms (1). It constitutes up to 50% of the total amino acids in tea and accounts for approximately 1–2% of its dry weight, contributing to tea's characteristic umami flavor (2, 3). The widespread consumption of tea, together with its long history of traditional use for supporting various aspects of wellbeing, has led to significant scientific interest in the effects of L-theanine on human health and performance (4, 5).

Following oral ingestion, L-theanine is rapidly absorbed with plasma concentrations increasing in a linear dose-dependent manner in humans, peaking approximately 45–50 min post-consumption (6, 7). Owing to its structural similarity to the neurotransmitters glutamate and glutamine, its ability to cross the blood–brain barrier, and its reported effects on other neurotransmitters and neural oscillations (8–10), a considerable body of research has focused on L-theanine's potential to modulate the central nervous system and related functions. This has also led to its inclusion in dietary supplements as well as functional food and beverage products for its purported relaxation, sleep-supporting and cognition-enhancing (“nootropic”) effects (11, 12).

In this article, we summarize the latest research on L-theanine and its effects on cognition, mood and sleep based on systematic reviews and meta-analyses of human randomized controlled trials. In addition, we examine evidence relating to its safety, consider the limitations of the current literature and highlight key areas for future research. This review was based on a literature search using the keyword “theanine” in PubMed and Google Scholar, covering publications up to and including June 2026.

## 2 Cognition

There is growing interest among consumers for products that can help to support aspects of cognitive performance, such as attention, decision-making and memory, as well as associated subjective measures like perceived alertness and mental fatigue. Recent meta-analyses of randomized controlled trials have indicated that a single dose of L-theanine administered as a standalone intervention can temporarily enhance aspects of cognitive function in healthy adults (13, 14). In a review of five placebo-controlled trials, Mátyus et al. (13) reported small but statistically significant beneficial performance effects in favor of L-theanine on processing speed-based measures derived from visual attention tasks, but not on more simple reaction time or more complex (and potentially executive function-taxing) measures. A separate review and meta-analysis of largely overlapping studies by Payne et al. (14) also noted broadly consistent effects, indicating L-theanine significantly enhanced choice reaction time and rapid visual information processing task performance, but had non-significant effects on measures of simple reaction time and digit vigilance task performance (see Table 1). While these findings are promising, they should be interpreted with caution given the small number of associated studies included in each of these analyses. Among the included trials, cognitive assessments were typically conducted 40–70 min after ingestion to align with anticipated peak L-theanine plasma concentrations. Dosing in these studies ranged from 50 mg to approximately 500 mg and were considered safe and well-tolerated with a side effect profile that did not differ from placebo (13). While there is currently insufficient evidence to establish optimal dosing levels, single doses of L-theanine in the 100–400 mg range have been reported to significantly enhance measures of cognitive performance compared to placebo. Direct assessments of dose–response effects within this range are also limited and inconsistent (15, 16), suggesting further investigation of the magnitude, dose-response relationships and potential moderators of these pro-cognitive effects are warranted. Studies included in these reviews have also largely focused on visual attention and/or reaction time based measures, consistent with the cognitive domains typically enhanced by caffeine administration (17). Future trials should also examine effects on a wider range of domains, such as different aspects of memory and executive function, to better understand the broader profile of potential cognitive effects. Positive effects among these additional cognitive domains have been reported in trials in which L-theanine has been administered in combination with other actives, suggesting further evaluation of its standalone effects are also warranted (18, 19). In addition, further assessment of L-theanine's effect on associated subjective measures, such as perceived alertness, would be useful.

**Table 1:** Key systematic reviews of randomized controlled trials assessing the effects of L-theanine on cognition, mood and sleep.

Publication + type of review	Objective	Date of literature search	Number of included studies	Number of participants	Key findings
Cognition					
Mátyus et al. (13) - meta-analysis	To evaluate the effect of L-theanine on cognitive functions	July 2024	RCT - *n* = 5 (all standalone intervention)	*n* = 148	L-theanine had a dose-dependent effect on cognitive function based on rapid visual information processing and recognition visual reaction time [MD = −15.20 ms, 95% CI (−28.99, −1.41); *n* = 5 studies]. The effects of L-theanine were non-significant on reaction time to a simple stimulus [MD = −0.46 ms, 95% CI (−15.65, 14.73); *n* = 5 studies] and on the Stroop test to both congruent [MD = −37.38 ms, 95% CI (−86.39, 11.62); *n* = 4 studies] and incongruent stimuli [MD = 109.28 ms, 95% CI (−8.72, 227.27); *n* = 4 studies]
Payne et al. (14) - meta-analysis	To evaluate the effect of tea, L-theanine alone, or L-theanine plus caffeine on cognitive functions	August 2023	RCT - *n* = 6 (all standalone intervention)	*n* = 147	There was a small-to-moderate difference in favor of L-theanine over placebo on choice reaction time in the first hour after intake [SMD = −0.35, 95% CI (−0.61, −0.10), *p* = 0.02; *n* = 4 studies], as well as dose-dependent effects on rapid visual information processing task performance based on reaction time [SMD = −0.17, 95% CI (−0.30, −0.04), *p* = 0.04; *n* = 2 studies] and accuracy measures [SMD = 0.17, 95% CI (0.10, 0.23), *p* = 0.02; *n* = 2 studies] in the second hour after intake. There were no other statistically significant effects, including on simple reaction time or digit vigilance task performance
Mood					
Moshfeghinia et al. (39) - narrative synthesis	To evaluate the effect of L-theanine on psychiatric symptoms in patients with mental disorders	June 2023	RCT - *n* = 11 (*n* = 6 standalone intervention; *n* = 5 administered in combination with other actives)	*n* = 563	While there is evidence that L-theanine supplementation reduced psychiatric symptoms more than placebo in some RCT's involving individuals with schizophrenia, anxiety disorders, and ADHD, the authors emphasized the need for further trials to validate these preliminary findings
Williams et al. (32) - narrative synthesis	To evaluate the effect of L-theanine on stress responses and anxiety levels	March 2019	RCT - *n* = 9 (*n* = 8 standalone intervention; *n* = 1 administered in combination with other actives)	*n* = 270	The authors concluded that supplementation with 200–400 mg/day of L-theanine may assist in the reduction of stress and anxiety in people exposed to stressful conditions
Sleep					
Bulman et al. (45) - meta-analysis	To evaluate the effect of L-theanine on sleep-related outcome measures	September 2024	RCT - *n* = 19 (*n* = 8 standalone intervention; *n* = 11 administered in combination with other actives)	*n* = 897	L-theanine significantly improved subjective sleep onset latency [SMD = 0.15, 95% CI (0.01, 0.29), *p* = 0.04; *n* = 10 studies], subjective daytime dysfunction [SMD = 0.33, 95% CI (0.16, 0.49), *p* < 0.001; *n* = 9 studies], and overall subjective sleep quality score [SMD = 0.43, 95% CI (0.04, 0.83), *p* = 0.03; *n* = 12 studies]. There were no significant differences between groups for other measures, including subjective and objective measures of sleep duration
Cotter et al. (44) - narrative synthesis	To evaluate the effect of L-theanine on sleep-related outcome measures	February 2025	RCT - *n* = 11; Single-arm, open-label trial - *n* = 2 (all standalone intervention)	*n* = 550	The authors concluded that supplementation with 200–450 mg/day of L-theanine appears to be a safe and effective way to support healthy sleep in adults. Results were most robust for markers of sleep quality, with little evidence that L-theanine increases total sleep duration

MD: Mean difference; RCT: Randomized controlled trial; SMD: Standardized mean difference.

There has also been significant interest in exploring potential interactive effects between L-theanine and caffeine, due to their natural co-occurrence in tea. Based on a series of meta-analyses, Payne et al. (14) concluded that this combination can enhance attentional task performance and potentially some aspects of mood, compared to placebo. However, this review did not attempt to disentangle the independent contributions of these actives by comparing this combination to the individual constituents administered in isolation. Indeed, consumption of caffeine alone is known to enhance both speed and accuracy-based measures of attention task performance (17). Individual studies have, however, suggested that co-ingestion of caffeine and L-theanine may have an additive effect, with additional benefits noted compared to when each is administered in isolation (20–27), although such findings have been somewhat inconsistent (28, 29) and would benefit from further behavioral and mechanistic evaluation. Though it is not clear if there is an optimal dose and/or ratio of caffeine to L-theanine to exert beneficial effects, to the best of our knowledge, all trials reporting additive effects have used a dose of L-theanine ≥100mg and a ratio of L-theanine to caffeine between 1:1 and 2:1. In addition to caffeine, emerging evidence indicates that L-theanine may also exert additional complementary pro-cognitive effects when consumed in combination with other dietary actives (18, 30), suggesting this is another area that warrants further exploration.

Overall, current evidence suggests that single doses of L-theanine can support healthy adults' cognitive performance, particularly on visual attention tasks and when co-administered with caffeine. Additional well-designed, adequately powered trials are needed to further evaluate the magnitude and profile of effects of L-theanine across a broader range of cognitive domains, establish optimal doses, better understand interactions with other co-consumed bioactive compounds (including caffeine), and to identify potential moderators of efficacy.

## 3 Mood

L-theanine's reported ability to modulate neurotransmitters has led to significant interest in its ability to influence aspects of mood, including stress and anxiety. Given the high prevalence of perceived stress in the general population (31), safe and effective nutritional supplements may offer a useful strategy for supporting wellbeing. The most comprehensive systematic review of participant-reported outcome measures and physiological stress response data included nine randomized placebo-controlled trials (*n* = 270) and concluded that supplementation with 200–400 mg/day of L-theanine may assist in the reduction of stress and anxiety in people exposed to stressful situations (32). The authors noted that L-theanine may exhibit beneficial properties during times of heightened acute stress or among individuals with a high propensity for stress and anxiety, potentially via the production of alpha-waves and a decrease of glutamate in the brain, though they also flagged that there is currently insufficient evidence to suggest it assists in the reduction of stress levels in people with chronic conditions (32). This review included mainly acute (*n* = 6) but also some repeat dosing studies (*n* = 3), the latter studies spanning 17 days to eight weeks of daily dosing. Seven of the eligible studies recruited participants with no known pre-existing mental health issues, while two studies recruited participants with an existing mental health condition. The authors of the review considered the included trials to have a generally “low” risk of bias across key aspects of trial methodology and reported that L-theanine was well-tolerated, exhibiting a lack of adverse events. The authors also highlighted the need for more studies, including further assessment of L-theanine's effects when consumed over an extended period and when consumed in combination with other bioactive ingredients.

Interestingly, subsequently published trials have reported results that are consistent with the findings of this earlier review. For example, a randomized, double-blind, placebo-controlled, crossover trial involving healthy Japanese adults (*n* = 30) reported that supplementation with 200 mg/day of L-theanine for four weeks was associated with a significant reduction in participant-reported mood and anxiety symptoms relative to baseline that did not occur during the placebo condition, though between group analyses showed only a trend-level difference in mean change scores between study arms (33). Another randomized trial conducted in healthy young adults (*n* = 120) has also reported statistically significant beneficial effects relative to placebo when L-theanine (200 mg) was administered either alone or in combination with L-arginine (50 mg) prior to an acute psychological stressor task on subsequent salivary alpha-amylase activity, a measure of physiological stress response (34). A small, randomized trial by Lim (35) (*n* = 18) has also provided further evidence of reduced subjective and physiological stress responses following acute L-theanine administration. During an archery competition, a real-world scenario that invokes stress and anxiety, competitors who received 200 mg of L-theanine had significantly lower salivary alpha-amylase and salivary cortisol levels as well as self-reported anxiety scores immediately prior to the competition than those who received placebo (35). However, inconsistent results in other studies involving healthy adult samples suggest further high-quality trials on this topic would be useful (36–38). Collectively, these additional studies assessing L-theanine's effects on stress and anxiety also indicate that an updated systematic review on this topic would be a valuable addition to the scientific literature.

A systematic review by Moshfeghinia et al. (39) evaluating the effects of L-theanine exclusively in patients with mental health disorders revealed that there are currently relatively few studies in such clinical populations. While there was evidence that L-theanine supplementation reduced psychiatric symptoms more than placebo in some randomized controlled trials involving individuals with schizophrenia, anxiety disorders, and ADHD, the authors stressed that caution is warranted in their interpretation, emphasizing the need for further well-designed trials to validate these preliminary findings (39). An earlier network meta-analysis of studies conducted in patients diagnosed with anxiety disorders reported that L-theanine did not outperform placebo in alleviating associated clinical symptoms, though this was based on data from only a single trial (40).

Overall, trials have indicated that supplementation with 200–400 mg/day of L-theanine can help to reduce both perceived and physiological markers of stress and anxiety in largely healthy adult samples, particularly during periods of acute stress. These studies have also reported this dosing range of L-theanine to be safe and well-tolerated. Importantly, unlike some relaxation aids, L-theanine does not appear to cause sedation or adversely impact cognitive function (41, 42), suggesting it may be appropriate for use during stressful moments at any time of day. However, there is currently insufficient evidence for L-theanine to be recommended as a standalone or adjunctive therapeutic intervention for chronic stress or any psychiatric conditions.

## 4 Sleep

Sleep is essential for our health and wellbeing, yet sleep problems are a common complaint among adults worldwide (43). Following evidence of L-theanine's ability to exert calming effects on the central nervous system, there has also been significant scientific interest in its potential to help support restful sleep. The two most recent and comprehensive reviews of trials that assessed the effects of L-theanine on sleep when used as a standalone intervention (44) or in combination with other active ingredients (45) have both concluded that it can support aspects of sleep quality.

In the most recent systematic review on this topic, our research group (44) concluded that supplementation with 200–450 mg/day of L-theanine, as a standalone intervention, appears to be a safe and effective way to support healthy sleep in adults based on data from 13 published trials (*n* = 550 participants). The results were most robust for markers of sleep quality, with little evidence that L-theanine increases total sleep duration. Associated beneficial effects were generally in the small-to-medium effect size range and were reported on both objective (e.g. actigraphy) and participant-reported outcome measures linked to sleep latency, maintenance and efficiency, as well as perceived sleep satisfaction and feelings of refreshment and recovery on waking. These conclusions were consistent with the findings from a separate review by Bulman et al (45) who pooled data from standalone intervention trials and those that administered L-theanine in combination with other active ingredients into a series of meta-analyses (19 trials, *n* = 897 participants). They reported that interventions containing L-theanine improved subjective measures of sleep onset latency, daytime dysfunction, and overall sleep quality scores to a significantly greater extent than comparator (largely placebo) conditions, with associated effect sizes in the small-to-medium range (see Table 1). No significant differences were found for other sleep measures, including sleep duration.

Collectively, these reviews included data from 22 randomized controlled trials, in which L-theanine was orally administered at doses ranging from 50–1,000 mg/day and over durations ranging from a single dose, to daily for eight weeks. Both reviews noted that L-theanine was well-tolerated with few and only mild adverse effects reported across the included studies. While improvements in sleep parameters were noted in healthy adults and various clinical populations, there has been limited evaluation of the efficacy of L-theanine in those meeting criteria for clinical insomnia, which represents an important area for future investigation. Both reviews also highlighted the need for more high-quality trials to assess the effects of L-theanine as a standalone intervention, ideally combining objective and participant-reported measures, to establish optimal intervention dosing, timing and duration and to identify potential moderators of its efficacy. To the best of our knowledge, we are unaware of the publication of any further randomized controlled trials in which L-theanine has been administered as a standalone intervention since the literature search conducted in Cotter et al. (44), though there are some relevant trials currently underway which should add important additional data to the current evidence base following their publication (46).

Overall, L-theanine has shown promise in randomized controlled trials as a safe and effective dietary supplement to help support sleep quality among adults in the general population. The optimal dosing protocol remains unclear, but supplementation with 200–450 mg/day seems to be effective based on studies administering L-theanine as a standalone intervention (and also aligns with the therapeutic range for its reported stress relieving effects). Further studies are required in those with clinical sleep disorders (including insomnia) before conclusions can be drawn with regards to its efficacy and suitability for use in such populations.

## 5 Discussion

Dietary supplementation with L-theanine has demonstrated a range of promising effects on key aspects of cognition, mood and sleep in human randomized controlled trials (summarized in Table 2). The strongest evidence to date supports its role in helping individuals cope with stressful situations and in improving sleep quality, however, there is also evidence that it can enhance aspects of cognitive performance (particularly when combined with caffeine). Effects have been reported across a range of participant-reported mood and sleep scales and associated physiological biomarkers, as well as performance-based tasks assessing cognitive function (particularly visual attention tasks). Consistent with its use and availability as a dietary supplement, the current evidence suggests L-theanine supports individuals with associated health complaints (e.g. difficulties with sleep, or experiencing a stressful event), but there is insufficient evidence to recommend L-theanine as a treatment for any associated clinical symptoms (32, 39, 40, 44).

**Table 2:** Summary of the current evidence from randomized controlled trials on the effects of L-theanine on cognition, mood and sleep in healthy adults.

Cognition	Mood	Sleep
100–400 mg can support aspects of cognitive performance (particularly during visual attention tasks) Effects seemingly strongest when co-consumed with caffeine – based on L-theanine dose ≥100mg and ratio of 1:1–2:1 (L-theanine:caffeine)	200–400 mg/day can help to reduce both perceived and physiological markers of stress and anxiety during stressful situations, without making users feel drowsy	200–450 mg/day can support various markers of sleep quality, without increasing total sleep time

L-theanine's beneficial effects appear to occur within a similar dose window (~100–400 mg), with slightly higher doses (≥200 mg) seemingly required to exert its stress-relieving and sleep enhancing effects compared to that reported to support cognitive performance (≥100 mg). Importantly, reviews of trials have consistently reported that this dose range appears to be safe and well-tolerated by participants, even over daily dosing for an extended period (13, 32, 39, 44, 45). While tea is the major source of L-theanine in the human diet, a standard 200 mL cup of commercially available green or black tea typically contains only around 8–25 mg of L-theanine, depending on the type of tea, manufacturing and preparation methods used (47, 48). This suggests that while some benefits may be achieved through routine tea consumption, additional L-theanine would likely need to be consumed to achieve its optimal effects, particularly given that caffeine is also present in many forms of tea and acts as a stimulant which can impact aspects of mood (both directly and indirectly via withdrawal) and sleep (49, 50).

L-theanine's ability to exert effects on cognition, mood and sleep suggests a complex interplay of underlying mechanisms. A series of prior reviews have provided detailed discussion of L-theanine's neurobiological effects (8–10), indicating that this appears to involve a range of processes, including modulation of neurotransmitters [including dopamine, serotonin, glycine, glutamate and gamma-aminobutyric acid (GABA)], neuroendocrine stress response systems and brain oscillatory activity. With regards to the latter, various trials have reported that single doses of L-theanine can increase alpha-waves in the brain in healthy adults with elevated stress/anxiety (36, 51–54). These are neural oscillations that are considered a marker of a relaxed but alert mental state (8). In addition, there is evidence that L-theanine may be able to buffer some of the adverse effects that can occur as a result of caffeine consumption, such as sleep disturbance and increases in anxiety (26, 55), consistent with findings reported in pre-clinical studies (56–58). Given the ubiquitous use of caffeine-containing products globally, often throughout the day, this could also have contributed to some of the observed beneficial effects.

Review of evidence across these three health domains highlighted the need for well-designed and appropriately powered trials of L-theanine supplementation to further examine the magnitude and profile of associated effects, as well as to establish optimal doses, the mechanisms underlying these benefits, evaluate potential interactions with other co-consumed bioactive compounds, and to identify moderators of efficacy. While statistically significant positive effects were reported across these domains, the small-to-moderate effect sizes noted in studies indicates a need for large samples to reliably detect such differences and may be a reason why smaller studies have reported some inconsistent findings. Though we centered this review on the most comprehensive (and therefore typically most recently published) systematic reviews of randomized controlled trials in these respective areas, it is important to note that there are also other reviews on these topics that cite consistent conclusions (42, 59–62). Finally, in addition to these positive effects identified for cognition, mood and sleep, there are various other emerging areas of research on L-theanine's potential physiological effects (2–5, 26, 27, 63), indicating that this plant-derived amino acid may provide a broad spectrum of benefits for human health and performance.
